# Perceptions of mandatory reporting among family physicians to protect victims of elder abuse in Mongolia

**DOI:** 10.1093/geroni/igaa057.3216

**Published:** 2020-12-16

**Authors:** Ariunsanaa Bagaajav

**Affiliations:** The Graduate Center, CUNY, New York, New York, United States

## Abstract

Every year, approximately 10% of older adults worldwide experience elder abuse (EA), a serious violation of human rights. A wide range of consequences of EA have been identified including increased vulnerability to morbidity and mortality. Some countries have regulations to protect victims of EA either under stand-alone EA laws or relevant laws such as mandatory reporting. As of 2016, family physicians (FPs) in Mongolia became mandatory reporters of domestic violence alongside other human service workers under the newly amended Law to Combat Domestic Violence. Evidence shows that health providers who report abuse cases have greater awareness of their mandatory obligations to report. No rigorous study has explored the extent to which FPs in Mongolia know about mandatory reporting and their perceptions about its effectiveness to help victims of EA. An exploratory qualitative study was conducted. Through purposive sampling, 15 FPs participated in semi-structured in-depth interviews. The data collection took place over Skype. All interviews were recorded, transcribed verbatim and analyzed using thematic analysis. Surprisingly, all participants were aware of their mandatory obligations to report cases of domestic violence including EA. However, the study revealed that FPs were doubtful that potential victims of EA would be effectively protected under the new regulation due to the public perception that services and legal resources are designed primarily for women and children. Addressing this misconception is critical for ensuring that resources are appropriately utilized for potential victims of EA as well.

